# Redox‐Responsive Polymeric Nanocapsules for Enhanced Tumor‐Targeted Delivery of Antimicrobial Peptides

**DOI:** 10.1002/advs.74480

**Published:** 2026-02-19

**Authors:** Lin Tang, Yajian Li, Xiaoyin Lv, Jianmei Guo, Yijia Zhang, Yuqi Lin, Kaili Nie, Jian Zeng, Ming Zhang, Qiong Dai

**Affiliations:** ^1^ Beijing Advanced Innovation Center for Soft Matter Science and Engineering Beijing University of Chemical Technology Beijing China; ^2^ College of Life Science and Technology Beijing University of Chemical Technology Beijing China; ^3^ Department of Medical Imaging Qilu Medical University Zibo China; ^4^ Department of Urology, National Cancer Center / National Clinical Research Center For Cancer / Cancer Hospital Chinese Academy of Medical Sciences and Peking Union Medical College Beijing China; ^5^ Beijing Key Laboratory of Urologic Cancer Cell and Gene Therapy, Ational Cancer Center / National Clinical Research Center for Cancer / Cancer Hospital Chinese Academy of Medical Sciences and Peking Union Medical College Beijing China; ^6^ Department of Pulmonary Surgery Hangzhou Institute of Medicine (HIM),Zhejiang Cancer Hospital Chinese Academy of Sciences Hangzhou China; ^7^ Department of Pathology Peking University International Hospital Beijing China

**Keywords:** antimicrobial peptides, cancer therapy, polymeric nanocapsule platform, redox‐responsive, targeted delivery

## Abstract

Antimicrobial peptides (AMPs) hold promise as anticancer agents but suffer from proteolytic instability, systemic toxicity, and hemolysis. Here, we report a modular redox‐responsive polymeric nanocapsule platform for enhanced tumor‐targeted AMP delivery. Using melittin (MEL) as a stringent model, disulfide‐crosslinked nanocapsules (nMEL) remain stable and inert under physiological conditions (<5% hemolysis) yet undergo glutathione‐triggered shell cleavage in the tumor microenvironment to reactivate lytic activity via controlled MEL liberation. nMEL exhibits ∼4‐fold higher tumor accumulation and suppresses subcutaneous tumor growth by ∼80% compared with free MEL. Incorporating phenylboronic acid ligands yields nMEL‐PBA, which actively targets hypersalivated tumor cells, achieving 4.5‐fold greater pulmonary enrichment and extending median survival to 43 days in a lung metastasis model. This strategy integrates systemic stability, tumor selectivity, and microenvironment‐responsive activation, providing a generalizable approach to overcome long‐standing barriers in AMP‐based cancer therapy.

## Graphical abstract

This study presents a modular redox‐responsive nanocapsule platform that enables enhanced tumor‐targeted delivery of antimicrobial peptides. Using melittin as an example, the system achieves controlled activation within the tumor microenvironment, improving intratumoral accumulation while minimizing systemic toxicity. In a lung metastasis model, the nanocapsules markedly limit metastatic burden and extend animal survival.

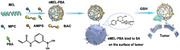

## 1 Introduction

Cancer remains one of the foremost causes of mortality worldwide, while conventional therapies‐including surgery, radiotherapy, and chemotherapy‐are often compromised by recurrence, metastasis, and drug resistance [1]. Antimicrobial peptides (AMPs) have recently emerged as a promising class of anticancer agents owing to their distinct mechanisms of action (Table S1) [2, 3]. These short amphipathic polypeptides (<100 amino acids) preferentially bind to negatively charged tumor cell membranes, perturb lipid packing, and induce rapid lytic death [4, 5]. Beyond direct cytotoxicity, AMPs also modulate antitumor immunity and inhibit angiogenesis, further broadening their therapeutic potential [6]. Notably, beyond oncology, AMPs are also intensively developed as anti‐infective agents; for example, AMP–photosensitizer conjugates have been reported to combat drug‐resistant biofilm infections via enhanced photodynamic therapy [7]. However, their clinical translation remains limited by proteolytic degradation, hemolysis, systemic toxicity, and unfavorable pharmacokinetics [8, 9].

To overcome these barriers, two complementary strategies have been pursued: rational peptide engineering and advanced nanodelivery. Sequence modifications can improve proteolytic stability and reduce off‐target toxicity, while nanocarriers enhance circulation, tumor selectivity, and stimulus‐responsive release (e.g., redox‐triggered activation in tumors [10]). Reported systems include dendrimers, liposomes, lipid nanoparticles, mesoporous silica, polymeric nanoparticles, and nanorods [11, 12, 13, 14, 15, 16, 17]. Representative examples highlight the potential of these approaches: a temperature‐responsive pNIPAm carrier that enables cooling‐triggered release of melittin at 25°C with effective tumor inhibition and minimal collateral damage [18], a dual pH/reactive oxygen species (ROS)‐responsive polymer that regulates KLA;(the model AMP) exposure and intracellular release to maximize efficacy while reducing systemic toxicity [19], and linoleic‐acid‐modified KLA that promotes self‐assembly, enhances antitumor activity, and mitigates hemolysis [20]. These studies underscore the promise of nanotechnology in conferring tumor selectivity, prolonged circulation, and spatiotemporal control. Yet, challenges remain, including limited in vivo stability, premature leakage, low loading capacity, synthetic complexity, and restricted applicability across peptide classes.

**FIGURE 1:**
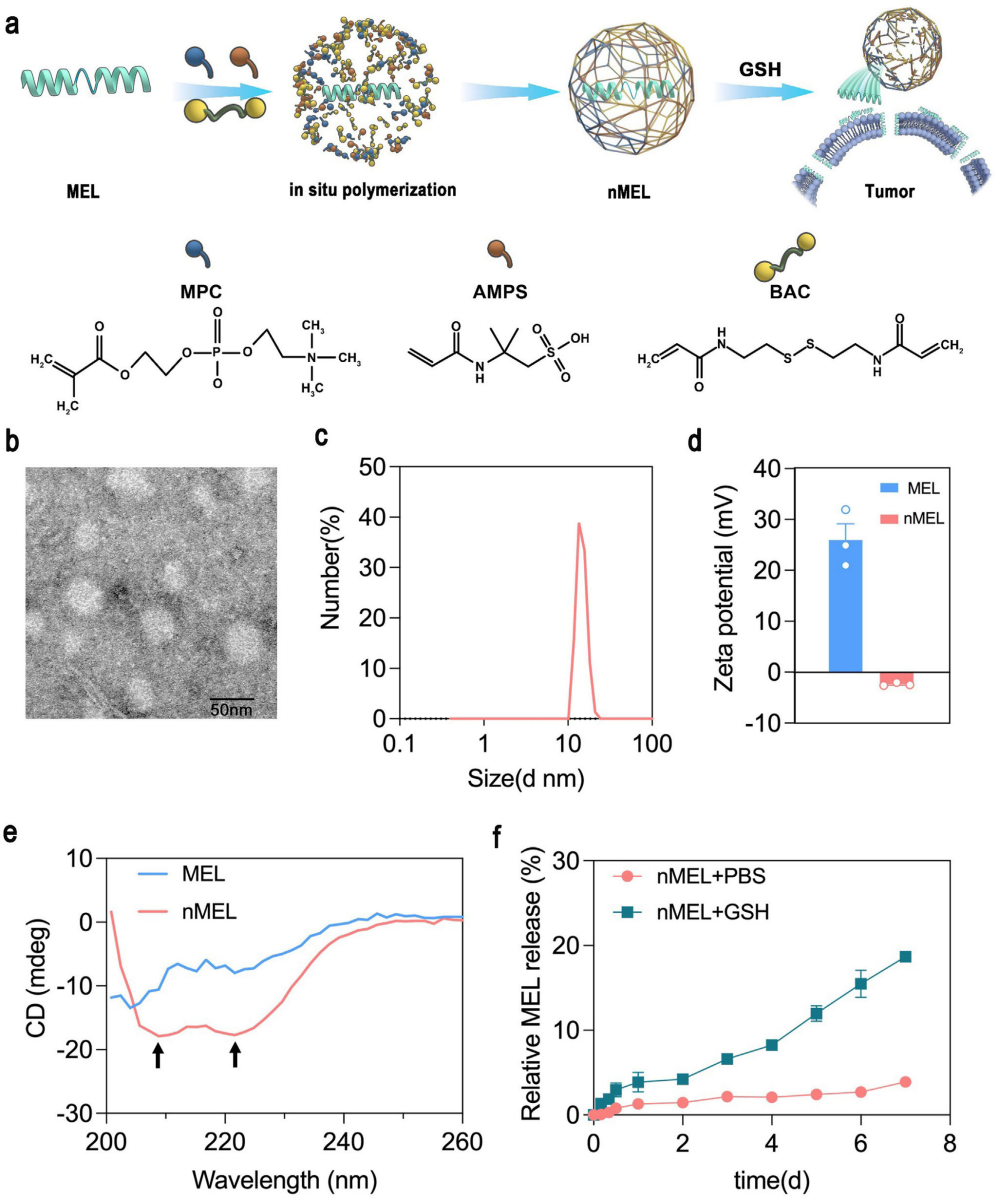
Synthesis and characterization of nMEL. (a) Schematic illustration of the in situ polymerization route and GSH‐triggered release mechanism. (b) TEM image, (c) hydrodynamic size distribution, and (d) zeta potential of nMEL. (e) Circular dichroism (CD) spectra of free MEL and nMEL, highlighting the characteristic α‐helical peaks at 208 and 222 nm (arrows). (f) Cumulative MEL release profiles from nMEL under simulated tumor microenvironment (20 µM GSH) and physiological PBS conditions. Data are shown as mean ± SD (*n* = 3).

Here, we report a modular redox‐responsive polymeric nanocapsule platform that enables systemic AMP delivery with enhanced tumor‐targeted activation. Melittin (MEL), a 26‐residue cationic peptide with potent antitumor activity but prohibitive hemolysis and systemic toxicity [21], was selected as a stringent model. In situ polymerization yields nanocapsules (nMEL) with a disulfide‐crosslinked shell that remains colloidally stable and functionally inert under physiological conditions (hemolysis <5%), yet undergoes glutathione (GSH)‐triggered cleavage in the reductive tumor microenvironment (TME) to restore MEL's lytic activity (Figure 1a). In vivo, nMEL significantly prolongs circulation, enhances tumor accumulation by ∼4‐fold compared with free MEL, and suppresses subcutaneous tumor growth by ∼80%. Moreover, incorporation of phenylboronic acid (PBA) ligands enables targeting of sialic acid (SA) overexpressing tumor cells, affording nMEL‐PBA with ∼4.5‐fold higher pulmonary tumor accumulation than nMEL, and extended median survival (43 days) in a 4T1 lung metastasis model (39 days for nMEL; 30 days for phosphate‐buffered saline (PBS)). Collectively, this strategy addresses key translational barriers by integrating systemic stability, enhanced tumor targeting, microenvironment‐responsive release, and provides a generalizable platform adaptable to diverse AMP cargos.

## 2 Results and Discussion

### 2.1 Synthesis and Characterization of nMEL

nMEL nanocapsules were synthesized by in situ polymerization and systematically characterized for size, charge, and protein conformation. Transmission electron microscopy (TEM) and dynamic light scattering (DLS) confirmed uniform spherical particles with an average hydrodynamic diameter of ~14 nm and a zeta potential of ~–3 mV (Figure 1b–d). By contrast, native MEL is highly cationic (∼+28 mV), and the pronounced shift to negative surface charge corroborates successful encapsulation. The incorporation of anionic monomers 2‐Acrylamido‐2‐methylpropanesulfonic acid (AMPS) imparts a net negative shell, promoting electrostatic complexation with cationic MEL during polymerization and thereby supporting high encapsulation efficiency. Circular dichroism (CD) further verified secondary‐structure preservation, with α‐helical signatures at 208 and 222 nm matching free MEL (Figure 1e), indicating that the encapsulation process maintains peptide integrity.

For therapeutic action, MEL must be liberated within tumors. We therefore probed redox responsiveness using a dialysis assay. In PBS, nMEL was highly stable with <4% cumulative release over 7 days, reflecting an intact, crosslinked shell that suppresses premature leakage (Figure 1f). To better reflect tumor extracellular redox conditions, we used 20 µM GSH, which lies within the reported micromolar extracellular range (≈2–20 µM) [22, 23]. Under these tumor‐mimicking reductive conditions (20 µM GSH), nMEL exhibited sustained release (∼20% / 7 d), consistent with disulfide cleavage of the BAC (N,N‐bis(acryloyloxy)cystamine) crosslinker in the shell and detectable reactivation of MEL bioactivity, even at a limited release fraction.

These data establish a functional dichotomy central to the design: colloidal stability and cargo sequestration under physiological conditions, coupled with redox‐triggered unsealing in a reductive milieu. The AMPS monomer‐mediated negative surface charge aids encapsulation and circulation compatibility, while the disulfide crosslinks provide a controllable gate for on‐demand release, together enabling minimal off‐target leakage yet activation in tumor‐relevant environments.

### 2.2 Cytotoxicity and Mechanism of nMEL

**FIGURE 2:**
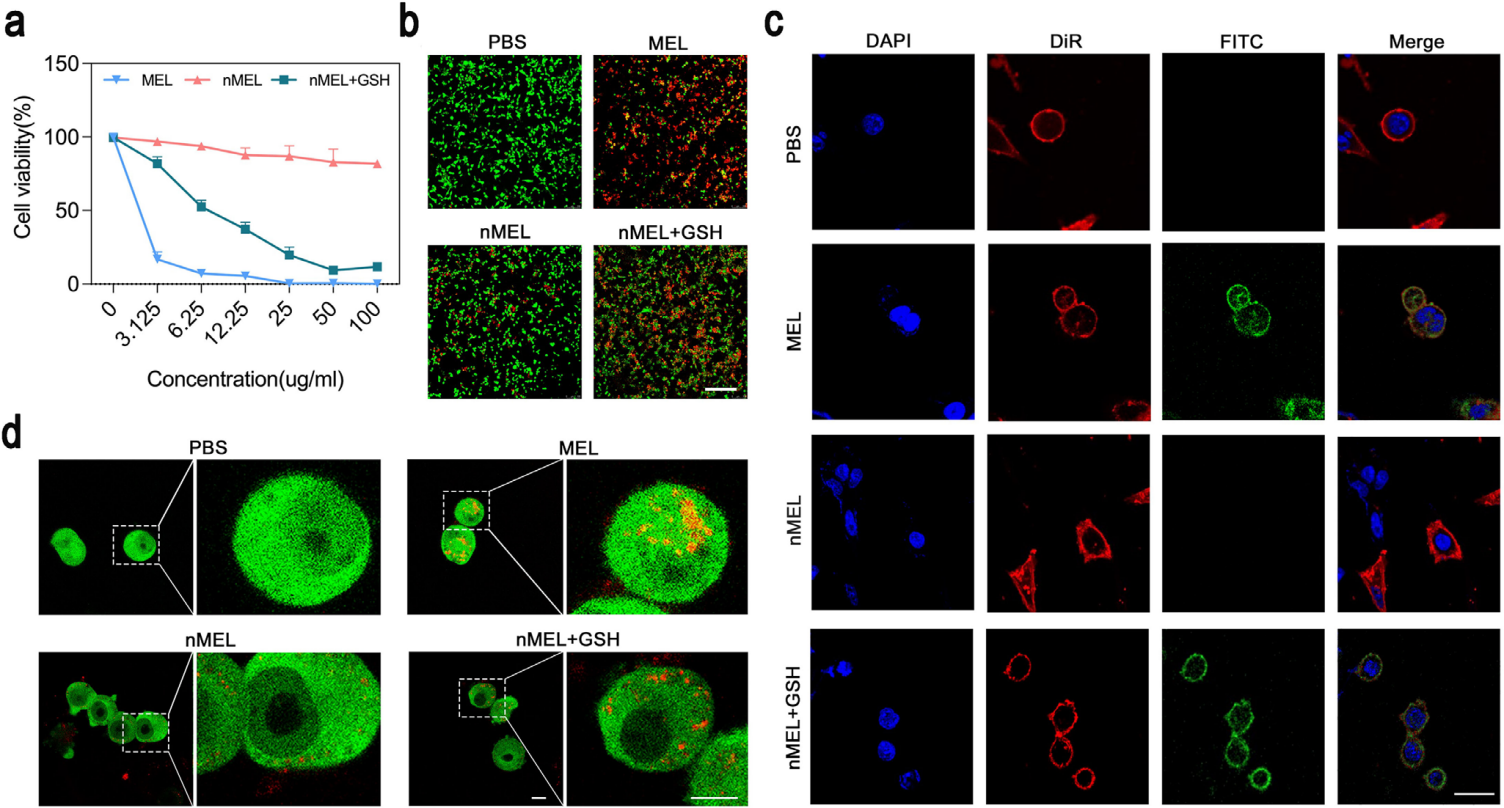
Cytotoxicity and mechanism of action of nMEL. (a) Viability of 4T1 cells after treatment with MEL, nMEL, or GSH‐pretreated nMEL (mean ± SD, *n* = 5). (b) Live/dead staining after 24 h incubation (MEL = 15 µg mL^−^^1^, scale bar: 100 µm). (c) Confocal images of 4T1 cells after 0.5 h co‐incubation with different formulations (PBS, MEL, nMEL, and nMEL+GSH), showing cell membranes (DiR, red) and FITC‐labeled MEL (green) (MEL = 5 µg mL^−^^1^, scale bar: 25 µm). (d) Confocal images of 4T1 cells after 4 h co‐incubation with different formulations (PBS, MEL, nMEL, and nMEL+GSH), with cytoskeleton stained by phalloidin (green) and MEL (labeled with Cy5.5, red) (MEL = 5 µg mL^−^^1^, scale bar: 25 µm).

MEL exerts potent cytotoxicity by electrostatically binding to negatively charged tumor cell membranes, inserting its hydrophobic domains, adopting an α‐helical conformation, and subsequently aggregating to form membrane pores that cause cell lysis and death [24, 25, 26]. Under our design, nMEL must first be activated by extracellular (tumor‐microenvironment–level) GSH to release MEL; the released MEL then adsorbs to the tumor cell membrane, perturbs membrane integrity, and can enter the cytosol to induce cell death. To evaluate whether nMEL preserves this activity in a redox‐dependent manner, we first examined its cytotoxicity in the well‐established 4T1 murine mammary tumor cell line. nMEL was pretreated with GSH for 12 h to mimic its behavior under reductive tumor microenvironment conditions, denoted as nMEL+GSH. As shown in Figure 2a, free MEL exhibited strong cytotoxicity with an IC_50_ of 0.83 µg mL^−^^1^. nMEL+GSH showed restored cytotoxicity relative to inert nMEL (IC_50_ = 8.05 µg mL^−^^1^), consistent with ongoing release of bioactive MEL after GSH pretreatment (12 h, ∼3% according to the release profile). Moreover, during cell co‐incubation, the nanocapsules can continue to release bioactive MEL, which helps explain why the cytotoxicity of 100 µg mL^−^^1^ nMEL+GSH is comparable to that of ∼3 µg mL^−^^1^ free MEL. These results indicate that bioactivity reactivation is driven by early/ongoing release, and a strict linear relationship between cumulative release percentage and cytotoxicity is not assumed. In contrast, untreated nMEL displayed negligible cytotoxicity, with >80% cell viability even at 100 µg mL^−^^1^, reflecting the lack of peptide release. Live/dead staining corroborated these findings, revealing extensive cell death in MEL and nMEL+GSH groups, whereas the nMEL group induced minimal toxicity (Figure 2b).

To probe the underlying mechanism, confocal imaging was performed using fluorescein isothiocyanate (FITC)‐labeled MEL. After 0.5 h of incubation with 4T1 cells, both MEL and nMEL+GSH localized to the cell membrane, indicative of the initial adsorption and pore‐forming process (Figure 2c). To further support this observation, colocalization analysis based on DiR/FITC double‐positive fluorescence line‐scans is provided in Figure S1. The MEL and nMEL+GSH groups display coincident DiR/FITC intensity peaks near the membrane region, whereas PBS and nMEL show negligible FITC signal and no apparent overlap. In contrast, untreated nMEL showed no detectable membrane association, confirming the absence of MEL release. To further assess longer‐term interactions, Cy5.5‐labeled MEL and nMEL were incubated with 4T1 cells for 4 h. Phalloidin staining of the actin cytoskeleton revealed pronounced cytoplasmic internalization of MEL and nMEL+GSH (red) in the presence of actin filaments (green), highlighting progressive membrane disruption and intracellular delivery (Figure 2d). By comparison, PBS and nMEL groups showed negligible MEL penetration.

These data indicate that nMEL is functionally inert under physiological conditions yet activates under reductive environments to liberate MEL, which retains its intrinsic membrane‐disruptive mode of action. The redox‐triggered release thus enables selective tumor cell killing while minimizing off‐target cytotoxicity in the absence of a reducing trigger.

### 2.3 In Vivo Biodistribution and Biosafety

**FIGURE 3:**
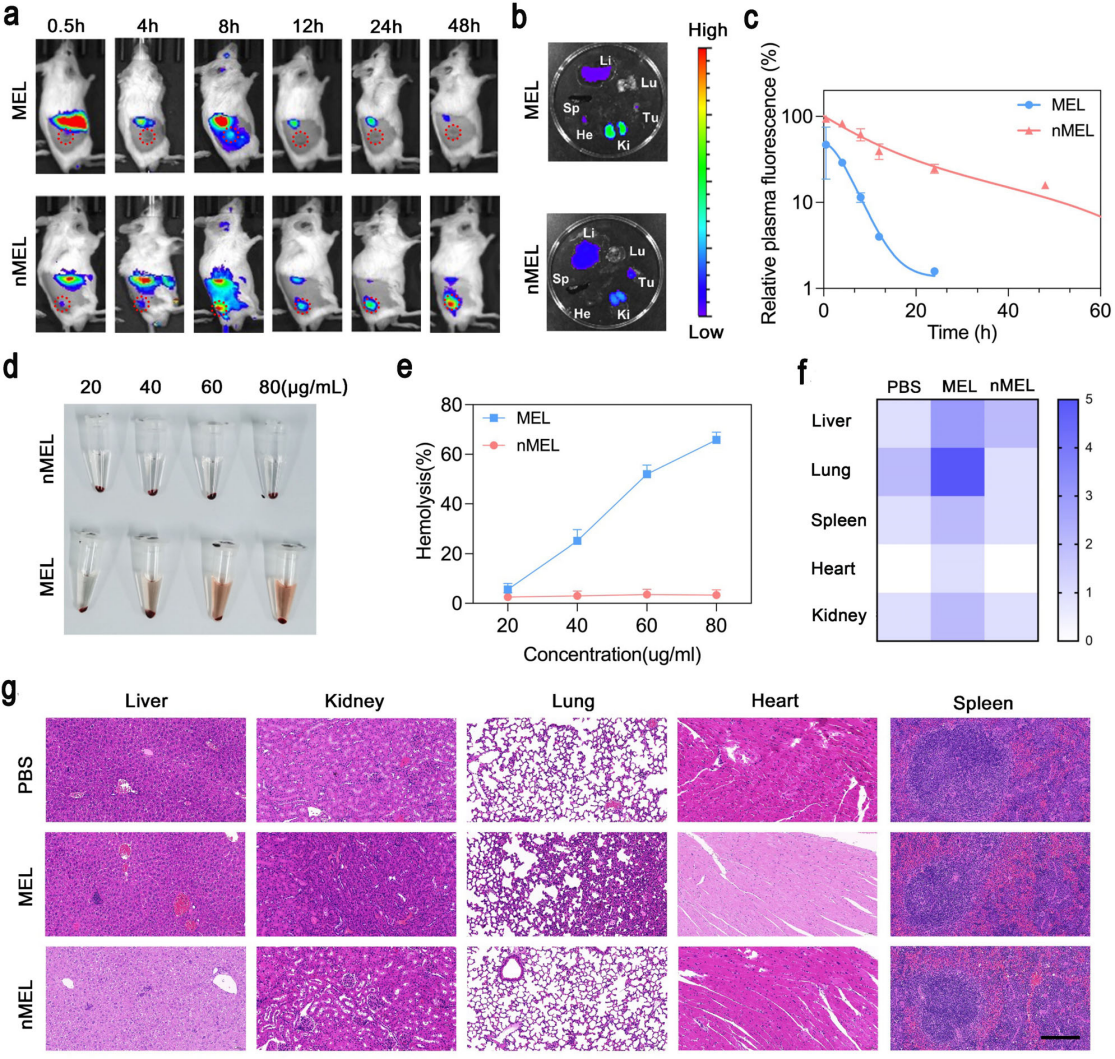
Biodistribution and biosafety of nMEL in the 4T1 subcutaneous tumor model. (a) In vivo fluorescence imaging after intravenous injection of Cy5.5 labeled‐MEL or nMEL (Ex/Em = 680/710 nm); tumor sites are indicated by red dashed lines. (b) Ex vivo fluorescence imaging of major organs at 48 h post‐injection. (c) Time‐dependent relative fluorescence intensity of MEL and nMEL in blood (mean ± SD, *n* = 3 mice per group). (d) Hemolysis assay of MEL and nMEL. (e) Hemolysis ratios at different concentrations (mean ± SD, *n* = 3). (f) Semi‐quantitative organ injury scores after 14 days of intravenous administration, evaluated by H&E staining (*n* = 3). (g) Representative H&E‐stained sections of major organs (scale bar: 200 µm).

To evaluate in vivo behavior and mechanism, Cy5.5‐labeled MEL and nMEL were administered to 4T1 subcutaneous tumor‐bearing mice. Whole‐body fluorescence imaging revealed sustained tumor accumulation of nMEL for up to 48 h, whereas free MEL was rapidly cleared from tumor sites within 8 h (Figure 3a). Ex vivo imaging at 48 h confirmed predominant hepatic/renal distribution for both cohorts; notably, tumor accumulation of nMEL was ≈4‐fold higher than free MEL (Figure 3b; Figure S2). Blood fluorescence quantification further showed prolonged circulation of nMEL, remaining detectable at 48 h, while free MEL became undetectable by 24 h (Figure 3c).

Enhanced tumor enrichment is consistent with: (i) extended systemic residence‐the ∼14 nm hydrodynamic size limits rapid renal filtration and the mildly negative surface (≈ –3 mV) dampens opsonization and reticuloendothelial system (RES) clearance; (ii) colloidal stability endowed by the disulfide‐crosslinked shell, suppressing premature peptide leakage so more intact carriers reach tumors; and (iii) the enhanced permeability and retention (EPR) effect, favoring deposition of long‐circulating nanocarriers within leaky tumor vasculature. Residual hepatic uptake aligns with expected recognition by the mononuclear phagocyte system. Importantly, the reductive tumor milieu cleaves disulfide linkages to liberate MEL, reconciling low off‐target toxicity in circulation with robust intratumoral activity. Together, these data establish that nanocapsule encapsulation couples pharmacokinetic extension with enhanced tumor accumulation and tumor‐preferential activation, yielding higher intratumoral exposure than free MEL while maintaining residual uptake in clearance organs.

Given MEL's non‐specific membrane‐lytic mode of action, hemolysis was first assessed by incubating graded concentrations of MEL or nMEL with diluted whole blood at 37°C for 1 h. As shown in Figure 3d,e and Figure S3, nMEL caused negligible erythrocyte damage (<5% hemolysis at 80 µg mL^−^^1^), in sharp contrast to the concentration‐dependent hemolysis induced by free MEL. This attenuation is consistent with charge shielding and steric confinement of MEL within the crosslinked shell, which limits peptide–membrane interactions under physiological conditions.

For systemic safety, major organs and blood were collected 14 days after intravenous administration. Histopathology (H&E) with semi‐quantitative injury scoring (Figure 3f,g) revealed organ‐specific toxicities in MEL‐treated mice: lungs showed alveolar septal widening, interstitial inflammatory infiltration, and focal collapse; livers displayed portal triad abnormalities with punctate necrosis and mild hepatocellular edema; kidneys exhibited glomerular depletion, atrophy, and blurred cortico‐medullary demarcation. In contrast, nMEL‐treated mice showed no abnormal tissue architecture, indistinguishable from PBS controls, and complete blood counts and hepatic/renal function indices remained within normal ranges (Table S2).

Encapsulation renders MEL functionally inert in circulation, reducing hemolysis and off‐target exposure, while redox‐triggered release in tumors restores peptide activity in situ. Consequently, nMEL decouples systemic safety from intratumoral potency, mitigates hemolytic and organ toxicities, and at the same time preserves on‐site efficacy, thereby offering superior biocompatibility and translational promise for in vivo antitumor therapy.

### 2.4 Antitumor Efficacy of nMEL in a 4T1 Subcutaneous Model

**FIGURE 4:**
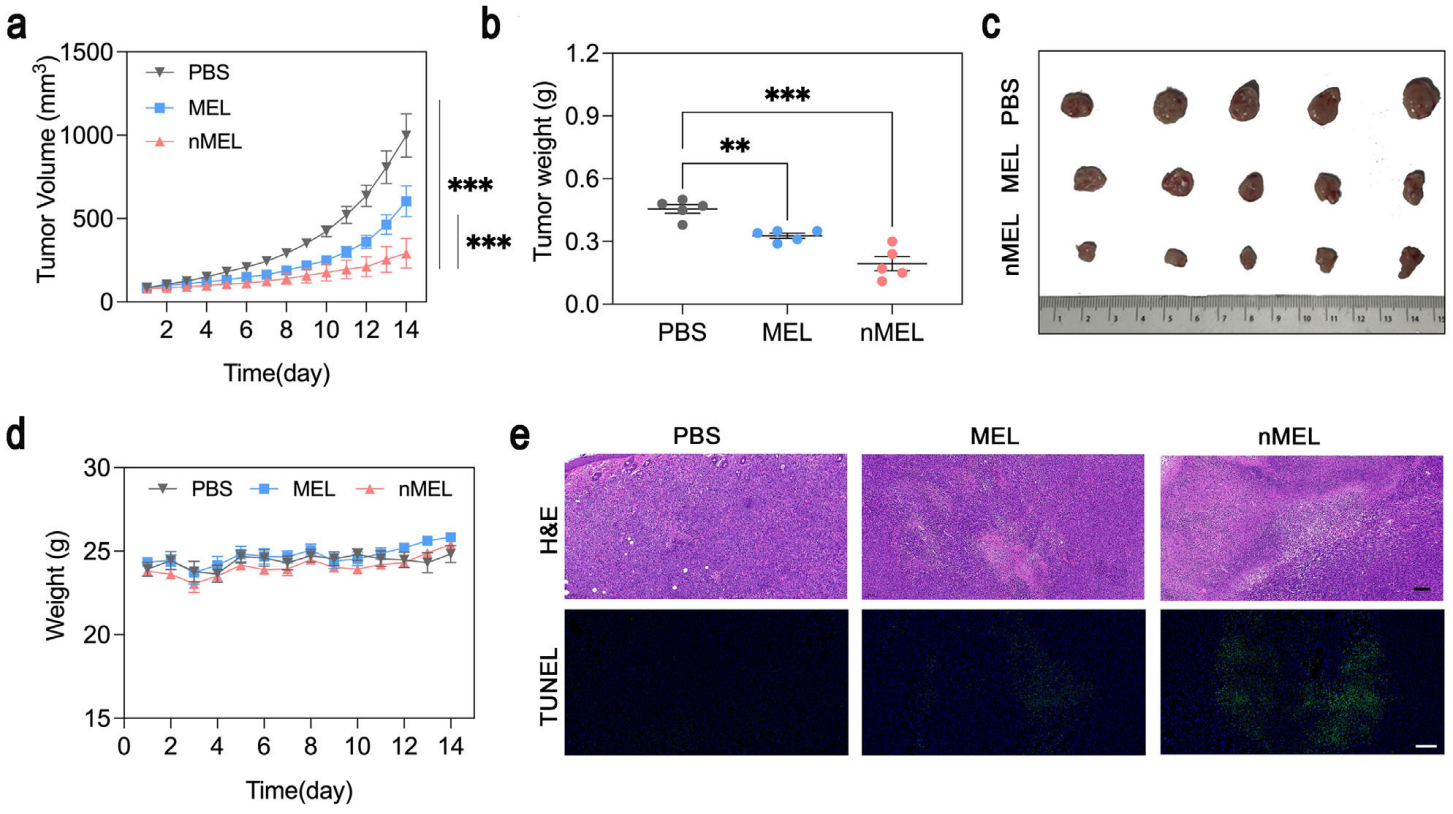
Antitumor efficacy of nMEL in the 4T1 subcutaneous tumor model. (a) Tumor growth curves of mice treated with PBS, MEL, or nMEL (mean ± SD, *n* = 5). Statistical significance was analyzed using two‐way ANOVA, ***p < 0.001. (b) Tumor weights at day 14 (mean ± SD, *n* = 5). Statistical significance was analyzed using one‐way ANOVA, **p < 0.01, ***p < 0.001. (c) Images of excised tumors from each group (*n* = 5). (d) Body weight profiles during treatment, confirming the absence of acute systemic toxicity (mean ± SD, *n* = 5). (e) Representative H&E‐ and TUNEL‐stained tumor sections after 14 days of treatment (scale bar: 100 µm).

Tumor‐bearing BALB/c mice (n = 5 per group) were randomized into three groups: PBS, free MEL, or nMEL. Intravenous dosing (MEL‐equivalent 2.5 mg kg^−^^1^) was performed on days 1, 3, 5, and 7, while body weight and tumor volume were monitored every two days. PBS controls exhibited rapid tumor progression, reaching approximately 1000 mm^3^ by day 14. Free MEL achieved moderate inhibition (∼600 mm^3^), whereas nMEL produced marked tumor control with terminal volumes of ∼200 mm^3^ (≈20% of PBS; Figure 4a). Excised tumor masses and gross images were consistent with these findings, confirming superior growth suppression by nMEL (Figure 4b,c). All groups maintained stable body weight, indicating the absence of acute systemic toxicity (Figure 4d). Histopathological analysis revealed preserved tumor architecture in PBS‐treated mice, focal necrosis following free MEL treatment, and extensive necrosis after nMEL administration (Figure 4e). In line with these results, terminal deoxynucleotidyl transferase dUTP nick end labeling (TUNEL) assays showed limited apoptosis in the MEL group, whereas nMEL‐treated tumors displayed extensive apoptotic signal.

The pronounced efficacy of nMEL can be attributed to its prolonged circulation and enhanced intratumoral accumulation (Figure 3), followed by redox‐triggered disulfide cleavage that releases bioactive MEL at the target site. This mechanism effectively separates systemic safety from on‐target potency: MEL remains sequestered and inert during circulation, thereby minimizing off‐target toxicity, but regains its membrane‐lytic activity within the reductive tumor microenvironment. The restored activity results in pronounced necrosis, apoptosis, and tumor volume reduction. Collectively, these results highlight that the polymeric nanocapsule platform addresses critical barriers to AMP translation, including circulation instability, hemolysis, and nonspecific toxicity, and establishes a viable strategy for harnessing membrane‐active peptides in oncology.

### 2.5 Design and Characterization of Actively Targeted nMEL‐PBA

**FIGURE 5:**
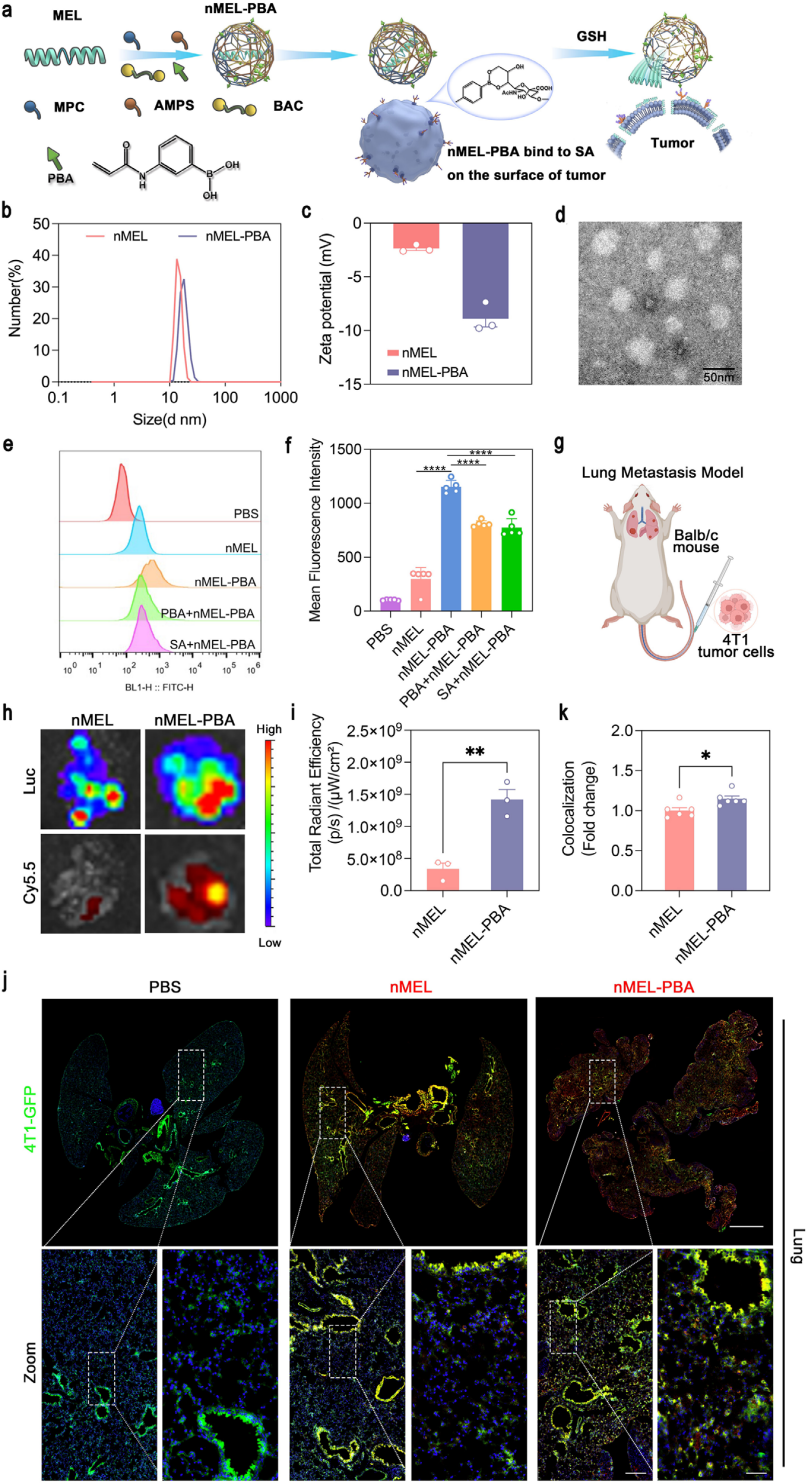
Characterization and validation of tumor‐targeting nMEL‐PBA nanocapsules. (a) Schematic illustration of nMEL‐PBA synthesis and targeting mechanism, in which PBA ligands bind to SA on tumor cell surfaces. (b) Particle size distribution, (c) zeta potential, and (d) TEM image of nMEL‐PBA. (e) Representative flow‐cytometry fluorescence distributions of FITC‐labeled formulations after incubation with 4T1 cells at 4°C for 4 h to suppress endocytosis and quantify surface association. Groups: PBS, nMEL, nMEL‐PBA, excess free PBA (10 mM, 30 min) pretreated cells + nMEL‐PBA, and free sialic acid (10 mM, 30 min) pretreated cells + nMEL‐PBA (MEL equivalent concentration: 15 µg mL^−^^1^). (f) Quantification of mean fluorescence intensity (MFI) from (e). Data are presented as mean ± SD (*n* =5). Statistical significance was determined by one‐way ANOVA, ****p < 0.0001. (g) Schematic of the mouse lung metastasis model established by intravenous injection of 4T1‐Luc cells. (h) Ex vivo fluorescence imaging of lungs after intravenous administration of Cy5.5‐labeled nMEL or nMEL‐PBA. (i) Quantitative fluorescence analysis of lung tumor signals based on (h) (mean ± SD, *n* = 3). Statistical significance was determined using one‐way ANOVA, **p < 0.01. (j‐k) Confocal images and quantitative fluorescence analysis showing co‐localization of Cy5.5‐labeled nanocapsules (red) with GFP‐expressing 4T1 tumor cells (green) in lung sections (mean ± SD, *n* = 6). scale bar: 2000, 500, and 150 µm. Statistical significance was determined using one‐way ANOVA, *p < 0.05.

In prior sections, redox‐responsive nMEL mitigated the side effects of free MEL and enhanced tumor accumulation through passive targeting. However, MEL's non‐specific membrane lysis necessitates active tumor recognition to improve cellular selectivity. In this context, a widely adopted strategy is to decorate nanocarriers with ligands that engage tumor‐associated receptors, thereby strengthening cell‐surface binding and facilitating receptor‐mediated uptake [27, 28]. Guided by these principles, we selected sialic acids (SA)—nine‐carbon acidic monosaccharides capping glycoproteins and glycolipids on vertebrate cell surfaces—as a tumor‐associated target [29]. Tumors frequently display hypersialylation, which correlates with malignant progression and poor prognosis [30, 31]. Phenylboronic acids form reversible covalent complexes with cis‐diols on SA, providing a chemistry handle for ligand‐directed targeting [32, 33]. We therefore introduced 3‐acrylamidophenylboronic acid (PBA) into the nMEL shell to generate nMEL‐PBA, aiming to enhance tumor cell binding while preserving redox‐triggered release (Figure 5a).

PBA was co‐polymerized using the same in situ procedure. Compared with nMEL, nMEL‐PBA displayed a modestly larger hydrodynamic diameter (∼34 nm) and a more negative zeta potential (−8.9 mV), attributable to PBA incorporation (Figure 5b–d). These physicochemical shifts, together with unchanged colloidal monodispersity, support successful ligand presentation on the nanocapsule surface. 4T1 is reported to exhibit upregulated sialic‐acid pathway activity compared with less metastatic related lines, consistent with increased capacity for cell‐surface sialylation [34]. We further performed a competitive cell‐surface binding assay under endocytosis‐suppressed conditions (4°C) using FITC‐labeled formulations to validate the specific PBA–SA recognition can enhance the tumor accumulation of nMEL‐PBA. As shown in Figure 5e,f and Figure S4, nMEL‐PBA produced a pronounced right‐shift in FITC fluorescence and significantly higher mean fluorescence intensity (MFI) compared with nMEL, indicating stronger surface binding. Importantly, competitive blockade with excess free PBA or free sialic acid markedly reduced the binding of nMEL‐PBA, as evidenced by the left‐shifted fluorescence distributions and decreased MFI values. These data provide direct mechanistic evidence that PBA–SA interactions contribute substantially to the improved tumor‐cell engagement of nMEL‐PBA.

### 2.6 Active Tumor Targeting in a 4T1 Lung Metastasis Model

A 4T1 lung metastasis model was established by tail‐vein injection of luciferase‐transfected 4T1 cells (4T1‐ Luc). Bioluminescence confirmed pulmonary colonization within 3–5 days (Figure 5g). Mice then received Cy5.5‐labeled nMEL or nMEL‐PBA intravenously, and organs were analyzed 3 days later. *Ex vivo* imaging revealed a strong luciferase signal in lungs, with markedly higher co‐localized Cy5.5 signal for nMEL‐PBA than for nMEL; quantitative analysis showed ∼4.5‐fold greater pulmonary accumulation for nMEL‐PBA (Figure 5h,i). Biodistribution across major organs is provided in Figure S5.

In a parallel cohort using green fluorescent protein (GFP)‐expressing 4T1 cells (4T1‐GFP), mice were dosed with Cy5.5‐labeled carriers, and lungs were sectioned 3 days post‐injection. Confocal microscopy showed greater colocalization of nMEL‐PBA (red) with GFP‐positive tumor foci (green) than nMEL, indicating improved cellular engagement within metastatic lesions (Figure 5j,k). Although some variability was observed among individual animals, the overall trend remained consistent and was supported by appropriate statistical analyses.

The enhanced pulmonary tumor localization of nMEL‐PBA is consistent with PBA–SA complexation on hypersialylated tumor cell surfaces, which augments adhesion and retention beyond EPR‐mediated deposition. The reversible covalent interaction is favored in mildly acidic tumor microenvironments and complements the disulfide‐mediated redox release of MEL, thereby coupling active recognition with on‐demand peptide activation. Together, these results demonstrate that PBA functionalization upgrades nMEL from passive to active targeting, improving delivery to metastatic pulmonary tumors while maintaining the platform's redox‐triggered mechanism.

### 2.7 Anti‐Tumor Effect of nMEL‐PBA on Lung Metastases

The goal of this study was to determine whether active sialic acid targeting by nMEL‐PBA could enhance survival and suppress pulmonary metastatic burden beyond the passive targeting of nMEL. To this end, an orthotopic 4T1‐Luc lung metastasis model was established by intravenous injection of 1 × 10^5^ cells into BALB/c mice (n = 11 per group, day 0). Mice were randomized into PBS, nMEL, or nMEL‐PBA groups. Intravenous treatments (2.5 mg MEL‐equivalent kg^−^^1^) were administered on days 1, 5, 10, and 15. Six mice per group were followed for survival, while five were used for quantification of metastatic nodules at the experimental midpoint.

**FIGURE 6:**
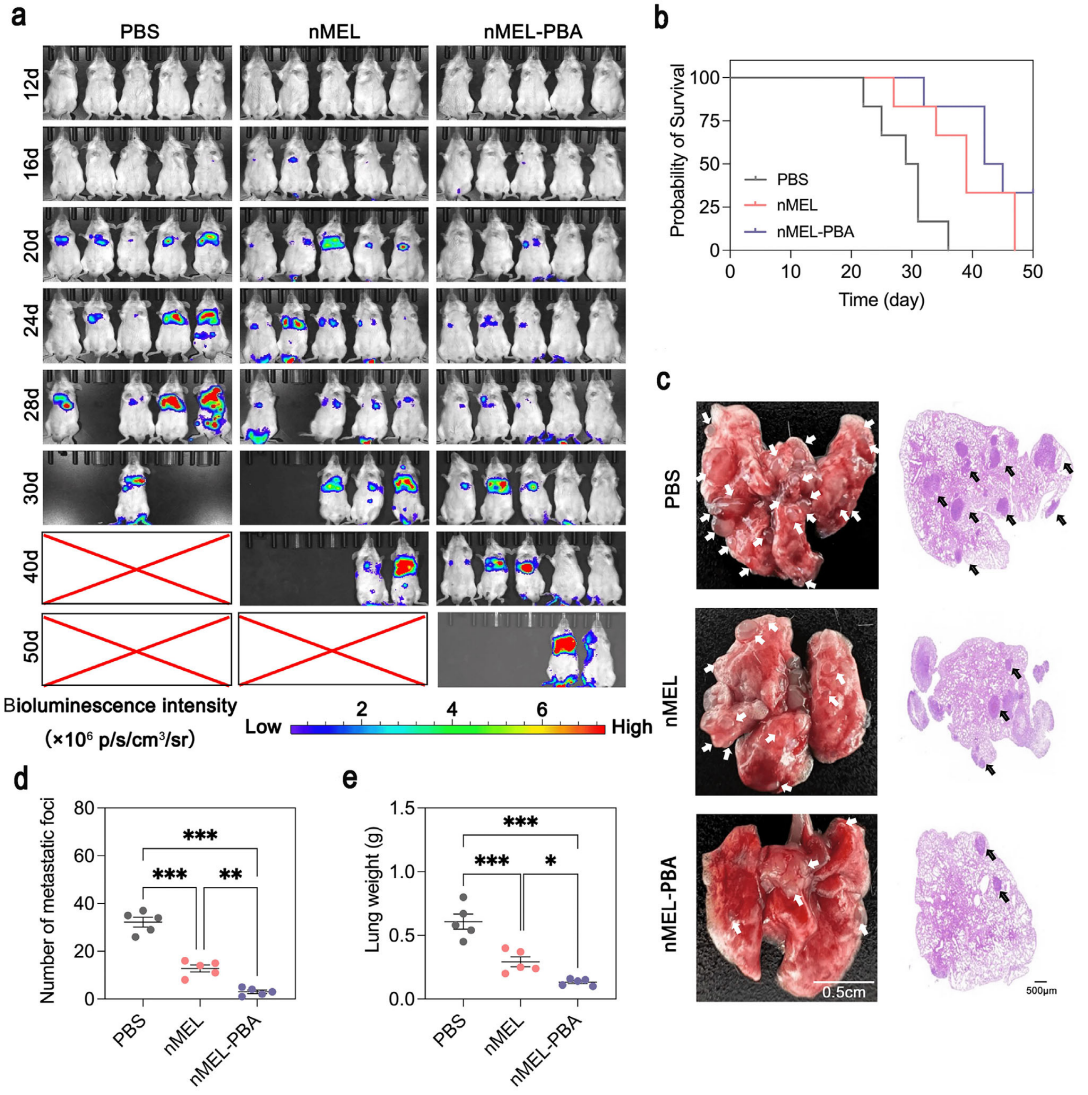
Antitumor efficacy of nMEL‐PBA in a 4T1 lung metastasis model. (a) Bioluminescence imaging showing pulmonary metastatic tumor burden at different time points (*n* = 6). (b) Kaplan–Meier survival curves of mice treated with PBS, nMEL, or nMEL‐PBA (*n* = 6). Statistical significance was analyzed using the log‐rank (Mantel–Cox) test (*p*=0.0011). (c) Representative images of isolated lungs (left) and H&E‐stained sections (right) on day 22; white arrows indicate metastatic nodules and black arrows denote metastatic lesions. (d) Quantification of metastatic lung nodules in each group (mean ± SD, *n* = 5). Statistical significance was determined using one‐way ANOVA, **p < 0.01, ***p < 0.001. (e) Lung weights in each group (mean ± SD, *n* = 5). Statistical significance was determined using one‐way ANOVA, *p < 0.05, ***p < 0.001.

Bioluminescence imaging beginning on day 12 revealed extensive lung metastases in PBS‐treated mice by day 20, whereas only faint signals were observed in the nMEL and nMEL‐PBA groups (Figure 6a,b). By day 40, all PBS mice had died, two nMEL mice survived, and five nMEL‐PBA mice remained alive. At day 50, two mice in the nMEL‐PBA group were still alive. Statistical analysis (Table S3) showed mean survival times of 29.00 ± 2.02 days for PBS, 38.83 ± 3.15 days for nMEL, and 43.50 ± 2.73 days for nMEL‐PBA. Median survival times were 30, 39, and 43 days, respectively. Lifespan extension was calculated as 30% for nMEL and 43.3% for nMEL‐PBA. These data demonstrate that both formulations significantly prolonged survival, with nMEL‐PBA conferring the greatest benefit.

To quantify lung colonization, mice were euthanized on day 22, and lung tissues were harvested for gross imaging and histopathology. PBS lungs exhibited dense tumor colonization with 32 ± 2.03 metastatic nodules, while nMEL reduced the number to 13 ± 1.46. Remarkably, nMEL‐PBA showed near‐complete inhibition, with only 3 ± 0.71 nodules (Figure 6c,d). H&E staining corroborated these findings. Consistently, lung weights paralleled metastatic burden, averaging 0.61 ± 0.06 g in PBS, 0.30 ± 0.04 g in nMEL, and 0.13 ± 0.01 g in nMEL‐PBA groups (Figure 6e), reflecting a dramatic reduction in overall tumor load.

 The pronounced therapeutic benefit of nMEL‐PBA arises from two synergistic design features. First, PBA–sialic acid interactions promote active tumor cell binding, thereby enhancing intrapulmonary accumulation beyond the passive EPR effect observed with nMEL. This active recognition is especially advantageous in disseminated microlesions where passive targeting is inefficient. Second, redox‐responsive disulfide cleavage within the tumor microenvironment restores the cytolytic activity of MEL locally, ensuring potent on‐site activity without systemic toxicity. Together, these mechanisms explain the reduced metastatic nodules, decreased lung mass, and significantly extended survival in nMEL‐PBA–treated mice. Collectively, these results establish that nMEL‐PBA not only prolongs survival but also effectively suppresses pulmonary colonization and metastatic proliferation, representing a promising strategy to overcome the translational barriers of AMP therapeutics in metastatic cancer.

## 3 Conclusion

In summary, we developed a redox‐responsive polymeric nanocapsule platform that enables efficient tumor delivery of AMPs while mitigating systemic toxicity, demonstrating broad translational potential. Using MEL as a model, nMEL exhibited 4‐fold higher tumor accumulation than free MEL. nMEL remained colloidally stable and inert under physiological conditions, yet underwent GSH‐triggered release in the tumor microenvironment to restore cytolytic activity and achieve potent tumor cell killing. In a subcutaneous 4T1 model, nMEL reduced terminal tumor volumes to ∼20% of controls. To further enhance targeting precision, PBA ligands were incorporated to generate nMEL‐PBA, which binds sialic acids overexpressed tumor cells. This active design increased pulmonary tumor accumulation by 4.5‐fold compared with nMEL and extended median survival to 43 days in a 4T1 lung metastasis model, significantly longer than nMEL (39 days) or PBS (30 days). Collectively, these findings establish nMEL and nMEL‐PBA as safe and effective nanotherapeutics. The modularity of this platform makes it broadly adaptable to diverse AMPs, providing a transformative strategy to advance peptide‐based cancer therapy.

## 4 Experimental Section

### 4.1 Synthesis of nMEL and nMEL‐PBA

The redox‐responsive nMEL were synthesized via in situ polymerization using: (1) MPC (2‐methacryloyloxyethylphosphorylcholine) as the biocompatible monomer; (2) AMPS (2‐acrylamido‐2‐methylpropanesulfonic acid) to enhance electrostatic interactions with MEL; and (3) BAC (N,N‐bis(acryloyloxy)cystamine) containing disulfide bonds as the redox‐sensitive crosslinker. MPC, AMPS, BAC, and initiators APS and TEMED were added to the MEL solution (MEL: MPC: AMPS: BAC: APS = 1:400:40:50:50, n/n), followed by in situ free radical polymerization. After reacting on ice for 2 h, unreacted MEL and monomers were removed by ultrafiltration. For the preparation of the tumor‐targeting nMEL‐PBA, PBA was additionally introduced into the solution (MEL: PBA = 1:40, n/n), while the remaining steps remained unchanged.

### 4.2 In Vitro Release of nMEL

FITC‐labeled MEL (MEL‐FITC) was encapsulated into nanocapsules (nMEL‐FITC). A dialysis device (100 kDa cutoff) was used: 1 mL of nMEL‐FITC (0.2 mg mL^−1^) was dialyzed against 5 mL PBS (with/without 20 µM GSH) at 37°C. Released MEL‐FITC in the outer solution was quantified by fluorescence (Ex/Em: 490/525 nm) at predetermined time points.

### 4.3 Hemolysis Assay

Whole blood was collected from Balb/c mice into heparin sodium‐coated tubes to prevent coagulation. The blood was diluted 1:1 with physiological saline (0.9% NaCl). nMEL and MEL were prepared at concentrations of 20, 40, 60, and 80 µg mL^−1^. Ultrapure water was used as the positive control, and commercially available physiological saline was used as the negative control. The samples were incubated at 37°C for 30 min. Subsequently, 60 µL of the diluted whole blood was added to each sample solution, and the mixtures were incubated at 37°C for 1 h. The samples were then centrifuged at 8000 rpm for 5 min, and the supernatant was collected. The absorbance of the supernatant was measured at 545 nm. The experiment was performed in triplicate.

$$\mathrm{Hemolysis}\;\left(\%\right)=\left({\mathrm{OD}}_{a}-{\mathrm{OD}}_{b}\right)/\left({\mathrm{OD}}_{c}-{\mathrm{OD}}_{b}\right)\;\times 100\%$$

where OD_a_​, OD_b_​, and OD_c_​ are the absorbances of the test sample, negative control, and positive control, respectively.

### 4.4 Colocalization of MEL With 4T1 cells

To investigate the adsorption of nMEL, MEL, and nMEL+GSH on the cell membrane of 4T1 cells, DiR (cell membrane dye) and FITC‐labeled samples were used. Add 500 µL of the DiR solution to each well and incubate at 37°C for 20 min. Wash the cells twice with 1× PBS to remove excess dye. nMEL, MEL, and nMEL+GSH solutions in serum‐free DMEM medium at a final MEL concentration of 5 µg mL^−1^ were added to Confocal dish, same steps as above. After 0.5 h, remove the solutions and wash the cells twice with 1× PBS. Wash the cells twice with 1× PBS to remove unbound samples. The results were observed under a confocal microscope. Further, the cytoskeleton was stained to explore the co‐localization of samples and cells after 4 h of co‐incubation. Cy5.5‐labeled nMEL, MEL, and nMEL+GSH solutions in serum‐free DMEM medium at a final MEL concentration of 5 µg mL^−1^ were added. After 4 h, remove the solutions and wash the cells twice with 1× PBS. Fix the cells with 4% paraformaldehyde for 15 min at room temperature. Phalloidin was added to each well and incubated at room temperature for 30 min in the dark. Wash the cells twice with 1× PBS to remove excess dye. The results were observed under a confocal microscope.

### 4.5 Cell‐Surface Binding Assay and Competitive Blocking

To quantify the cell‐surface association of nMEL‐PBA and verify the contribution of PBA–sialic acid recognition, an endocytosis‐suppressed flow cytometry assay was performed using FITC‐labeled formulations. FITC‐labeled MEL was first prepared and subsequently used to synthesize FITC‐labeled nMEL and nMEL‐PBA following the same procedures as the unlabeled formulations. 4T1 cells were seeded in 12‐well plates at 3 × 10^5^ cells per well and cultured to the desired confluence. For the competition groups, cells were preincubated with free PBA (10 mM) or free SA (10 mM) in serum‐free DMEM for 30 min. After pretreatment, FITC‐labeled samples (nMEL or nMEL‐PBA) in serum‐free DMEM at a final MEL‐equivalent concentration of 15 µg mL^−^^1^ were added and co‐incubated with cells at 4°C for 4 h to inhibit endocytosis and allow surface binding. The samples were then removed, and the cells were washed 2–3 times with cold 1× PBS to remove unbound formulations. Cells were collected and resuspended in 1× PBS for flow cytometry measurement. The mean fluorescence intensity (MFI) of the FITC channel was quantified using FlowJo V10.

### 4.6 Animal Experiment

Establishment of the 4T1 Subcutaneous Tumor Model: Balb/c mice (Female, 6‐8 weeks old) were subcutaneously injected with 4T1 cells (100 µL, 5 × 10^6^ cells per mouse). Tumor growth was monitored daily, and experiments were initiated when the tumor volume reached approximately 100 mm^3^. Establishment of the 4T1 Lung Metastasis Model: Balb/c mice (Female, 6–8 weeks old) were intravenously injected via the tail vein with 4T1‐Luc or 4T1‐GFP cells (100 µL, 1 × 10^5^ cells per mouse). After 3–5 days, tumor formation in the lungs of mice injected with 4T1‐Luc cells was visualized through bioluminescence of Luc by in vivo bioluminescence imaging.

### 4.7 Statistical analysis

All quantitative data are presented as mean ± SD unless otherwise stated. The number of biological replicates (n) and the specific statistical tests used are indicated in the corresponding figure legends. Statistical analyses were performed using GraphPad Prism 8.0.1. Survival curves were plotted using the Kaplan–Meier method and compared using the log‐rank (Mantel–Cox) test. The percentage increase in life span was calculated according to the following equation:

$$\mathrm{ILS}\;\left(\%\right)=\left({\mathrm{MST}}_{\mathrm{treated}}-{\mathrm{MST}}_{\mathrm{control}}\right)\;/\;{\mathrm{MST}}_{\mathrm{control}}\;\times 100$$

where MST_treated_is the median survival time of the treated group and MST_control_is the median survival time of the control group (derived from Kaplan–Meier survival analysis).

## Conflicts of Interest

The authors declare no conflicts of interest.

## Supporting information




**Supporting File**: advs74480‐sup‐0001‐SuppMat.docx.

## Data Availability

The data that supports the findings of this study are available in the supplementary material of this article.
