# Supplementary material for: Redox‐Responsive Polymeric Nanocapsules for Enhanced Tumor‐Targeted Delivery of Antimicrobial Peptides
Source: Adv Sci (Weinh). 2026 Feb 19;13(24):e74480. doi: 10.1002/advs.74480 (PMC13116077; doi:10.1002/advs.74480)
Supplement: Supplementary file 1 — Supporting File: advs74480‐sup‐0001‐SuppMat.docx. [file ADVS-13-e74480-s001.docx]

Supporting Information

Redox-Responsive Polymeric Nanocapsules for Enhanced Tumor-Targeted Delivery of Antimicrobial Peptides

Lin Tang, Yajian Li, Xiaoyin Lv, Jianmei Guo, Yijia Zhang, Yuqi Lin, Kaili Nie, Jian Zeng^*^[
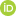
](Http://orcid.org/0000-0003-1008-4749), Ming Zhang^*^[
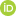
](Http://orcid.org/0000-0001-5841-1636), Qiong Dai ^*^[
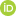
](https://orcid.org/0000-0003-1510-6054)

L. Tang, X. Lv, Q. Dai

Beijing Advanced Innovation Center for Soft Matter Science and Engineering
Beijing University of Chemical Technology
Beijing 100029, China

E-mail: daiqiong@mail.buct.edu.cn; ORCiD ID: 0000-0003-1510-6054

L. Tang, J. Guo, Y. Zhang, K. Nie, Q. Dai

College of Life Science and Technology
Beijing University of Chemical Technology
Beijing 100029, China

L. Tang

Department of Medical Imaging
Qilu Medical University
Zibo 255300, China

Y. Li

Department of Urology
National Cancer Center / National Clinical Research Center for Cancer / Cancer Hospital
Chinese Academy of Medical Sciences and Peking Union Medical College
Beijing 100021, China

Y. Li

Beijing Key Laboratory of Urologic Cancer Cell and Gene Therapy
National Cancer Center / National Clinical Research Center for Cancer / Cancer Hospital
Chinese Academy of Medical Sciences and Peking Union Medical College
Beijing 100021, China

Y. Lin, J. Zeng

Department of Pulmonary Surgery Hangzhou Institute of Medicine (HIM)

Zhejiang Cancer Hospital
Chinese Academy of Sciences
Hangzhou 310022, China

E-mail: zengjian@zjcc.org.cn; ORCiD ID: 0000-0003-1008-4749

M. Zhang

Department of Pathology
Peking University International Hospital
Beijing 102206, China

E-mail: [zhangming1@pkuih.edu.cn](mailto:zhangming1@pkuih.edu.cn); ORCiD ID: 0000-0001-5841-1636

1. **Materials**

Melittin (MEL, 2840 Da) was purchased from Chengdu Yunxi Chemical Co., Ltd. (China). 2-Methacryloyloxyethyl phosphorylcholine (MPC, ≥98%), 2-Acrylamido-2-methylpropanesulfonic acid (AMPS, 98%), Ammonium persulfate (APS, 98.0%) and tetramethylethylenediamine (TEMED, >99.0%) were purchased from HEOWNS. (China). N,N-Bis(acryloyl)cystamine (BAC, 98%) was obtained from Sigma-Aldrich (USA). 3-Acrylamidophenylboronic acid (PBA, 97%) was purchased from Aladdin Biochemical Technology Co., Ltd. (China). Phosphate-buffered saline (PBS), BCA Protein Assay Kit, and Cell Counting Kit-8 (CCK8) were purchased from Solarbio Life Sciences (China). Phosphotungstic acid (99.995%) and glutathione (GSH, 98%) were obtained from Sigma-Aldrich (USA) and Aladdin Biochemical Technology Co., Ltd. (China), respectively. NHS-Fluorescein Isothiocyanate (FITC), Cy5.5 NHS ester (98%) was purchased from YEASEN (China). 0.25% trypsin, DMEM Medium, Fetal Bovine Serum (FBS), Penicillium-streptomycin Mixture (PS) were purchased from Gibco (USA)

1. **Instrument**

Transmission Electron Microscopy (TEM, JEM-1200EX, JEOL Ltd., Japan) was used to observe the morphology of nanocapsules. Particle size and zeta potential were measured using Dynamic Light Scattering (Malvern Instruments, UK). Circular Dichroism (CD) spectra were recorded using a J-815 CD spectrometer (JASCO, Japan). A UV-Vis spectrophotometer (NanoDrop One, Thermo Fisher Scientific, USA) was utilized to determine the absorbance. Fluorescence intensity was measured using a multifunctional microplate reader (EnSpire, PerkinElmer, USA). An IVIS imaging system (Spectrum, PerkinElmer, USA) was applied for living image. Histological sections were prepared using a paraffin microtome (RM 2135, LEICA, Germany) and stained with hematoxylin and eosin (H&E). TUNEL staining was performed using a TUNEL assay kit (Roche) and imaged using a fluorescence microscope (BK-FL4, Chongqing Optics). Blood biochemical analysis was performed using an automatic biochemical analyzer (BS-420, Shenzhen Mindray).

1. **Characterization of nanocapsules**

The nanocapsules (nMEL or nMEL-PBA) were diluted to 1 μg/mL in PBS. A 10 μL sample was dropped onto a copper grid (200 mesh) and air-dried. After staining with 1% phosphotungstic acid for 50 s, the grid was analyzed using TEM at 100 kV.

nMEL or nMEL-PBA were diluted to 0.5 mg/mL in 0.1 M PBS (pH 7.2–7.4). Particle size and zeta potential were measured using DLS.

MEL and nMEL solutions (1 mg/mL) were prepared in PBS. CD spectra (200–260 nm) were recorded using a J-815 spectrometer with a 1 mm path length quartz cuvette.

1. **Synthesis of fluorescently labeled samples**

Firstly, the MEL was dissolved and dialyzed against PBS buffer (10 mM, pH 7.4) to remove the stabilizer that existed in the product powder. After dialysis, the MEL was diluted to 5 mg/mL with PBS. FITC-NHS and Cy5.5-NHS were dissolved with DMSO to make 10% (m/v) stock solutions, respectively. Subsequently, FITC-NHS and Cy5.5-NHS were added into MEL solutions at a molar ratio of 5:1 (Dye to MEL), respectively. The reactions were kept at 4 °C for 4 h and then were dialyzed thoroughly in PBS buffer to remove the dissociative dye.

1. **Cell Culture**

Mouse breast cancer cells (4T1, purchased from Wuhan Xavier Biotechnology Co., LTD) were cultured in DMEM medium supplemented with 10% fetal bovine serum (FBS) and 1% penicillin-streptomycin (PS) at 37°C under 5% CO₂.

1. **Cytotoxicity Assay**

4T1 cells were seeded in 96-well plates (1×10⁴ cells/well). After 24 h, cells were treated with nMEL, MEL (3.13, 6.25, 12.5, 25, 50, 100 μg/mL) for 24 h, where nMEL+GSH represents nMEL pretreated with 20μM GSH for 12h. Cell viability was assessed using CCK-8 reagent, and absorbance was measured at 450 nm.

Besides, live/dead staining was also used to evaluate the cytotoxicity of nMEL, MEL, and nMEL+GSH on 4T1 cells. 4T1 cells were seeded in a Confocal dish at a density of 2×10^6^ cells/well in DMEM complete medium (10% FBS, 1% PS), and incubated at 37°C with 5% CO₂ for 24 h. Then, nMEL, MEL, and nMEL+GSH solutions in serum-free DMEM medium at a final MEL concentration of 15 μg/mL were added to Confocal dish, respectively. After 24 h, remove the solutions and wash the cells twice with 1× PBS. Add 500 μL of the staining solution (Calcein-AM/PI) to each well and incubate at 37°C for 15–20 min in the dark. The results were observed under a confocal microscope.

1. **Living Imaging and Tissue Distribution of nMEL**

Balb/c mice (Female, 6-8 weeks old, purchased from Beijing Vital River Laboratory Animal Technology Co., Ltd.) were subcutaneously injected with 4T1 cells (100 μL, 5×10^6^ cells per mouse). Tumor growth was monitored daily, and experiments were initiated when the tumor volume reached approximately 100 mm³. Cy5.5-labeled samples (MEL, 2.5mg/kg) were intravenously injected into tumor-bearing mice. Live imaging and tail tip blood were performed at 0.5, 4, 8, 12, 24, 48 hours. After 48 hours, the mice were killed and the tumors of the heart, liver, spleen, lung and kidney were imaged.

1. **Antitumor Effect of nMEL**

Tumor-bearing mice were randomly divided into three groups (n=5): PBS, MEL, and nMEL. Intravenous administration (MEL 2.5 mg/kg) was performed on days 1, 3, 5, and 7, with body weight and tumor volume recorded every two days. After 14d, mice were sacrificed, tumor and organ tissues were collected for hematoxylin-eosin (HE) staining and TUNEL staining. At the same time, mouse heart blood was collected for the analysis of biochemical indexes and liver and kidney indexes.

1. **The targeting effect of nMEL-PBA on lung metastases tumor**

Balb/c mice (Female, 6-8 weeks old) were intravenously injected via the tail vein with 4T1-Luc (100 μL, 1×10^5^ cells per mouse). After 5 days, Cy5.5-labeled nMEL and nMEL-PBA were injected (n=3). 3 days later, living image was used to observe the co-localization of nMEL and nMEL-PBA with lung metastatic tumors.

In addition, 4T1-GFP cells were intravenously injected to establish the lung metastasis model. After 5 days, Cy5.5-labeled nMEL and nMEL-PBA were injected (n=3). 3 days later, the mice were euthanized, and lung tissues were collected for cry sectioning to observe the co-localization of nanocapsules with lung metastatic tumor cells.

1. **Survival analysis and Tumor Metastasis Evaluation**

The mice were randomly divided into three groups: PBS, nMEL, and nMEL-PBA (n=11) to establish the lung metastasis model. Among them, six mice were used for survival analysis, and five were used for counting lung metastatic nodules. On day 0,

4T1-Luc cells (1×10^5^ cells per mouse) were intravenously injected, followed by intravenous administration (MEL 2.5 mg/kg) on days 1, 5, 10, and 15. For survival analysis, living imaging was performed starting from 12d post-modeling to 50d. During the survival study, the number of surviving mice in each group was recorded, and living image was performed at regular intervals. On day 22, five mice from each group were euthanized, and lung tissues were collected to count metastatic nodules and measure lung weight. Subsequently, HE staining was performed on lung tissue sections to observe tumor metastasis. Survival analysis was performed using the Kaplan–Meier method, and differences among groups were assessed using the log-rank (Mantel–Cox) test.

**Table S1.** Representative Antitumor Antimicrobial Peptides

| *Name* | *Source* | *Mechanism of Action* | *Ref* |
| --- | --- | --- | --- |
| *Melittin* | Apis mellifera (honeybee) | Inhibits EGFR/HER2 phosphorylation; induces rapid membrane lysis | 1 |
| *MP9* | Engineered from mastoparan | Membrane disruption; immunogenic cell death; synergizes with PD-1/PD-L1 blockade | 2 |
| *TP4* | Nile Tilapia | ↑ROS; mitochondrial apoptosis (BAX/BCL-2); DNA damage | 3 |
| *CecropinXJ* | Bombyx mori (silkworm) | Membrane permeabilization; interacts with phosphatidylserine (PS) and phosphatidylethanolamine (PE) | 4 |
| *KLA* | Synthetic pro-apoptotic peptide | Mitochondrial membrane disruption; apoptosis | 5 |
| *Gramicidin S* | Bacillus brevis (bacterium) | Inhibits migratory capacity and G2/M cell cycle | 6 |
| *EAMPs* | Computational design | DNA damage; S/G2 cell cycle arrest; mitochondrial apoptosis | 7 |
| *Spider/Horseshoe crab peptides* | Brazilian spider / Japanese horseshoe crab | Rapid membrane lysis; kills dormant/resistant cells | 8 |


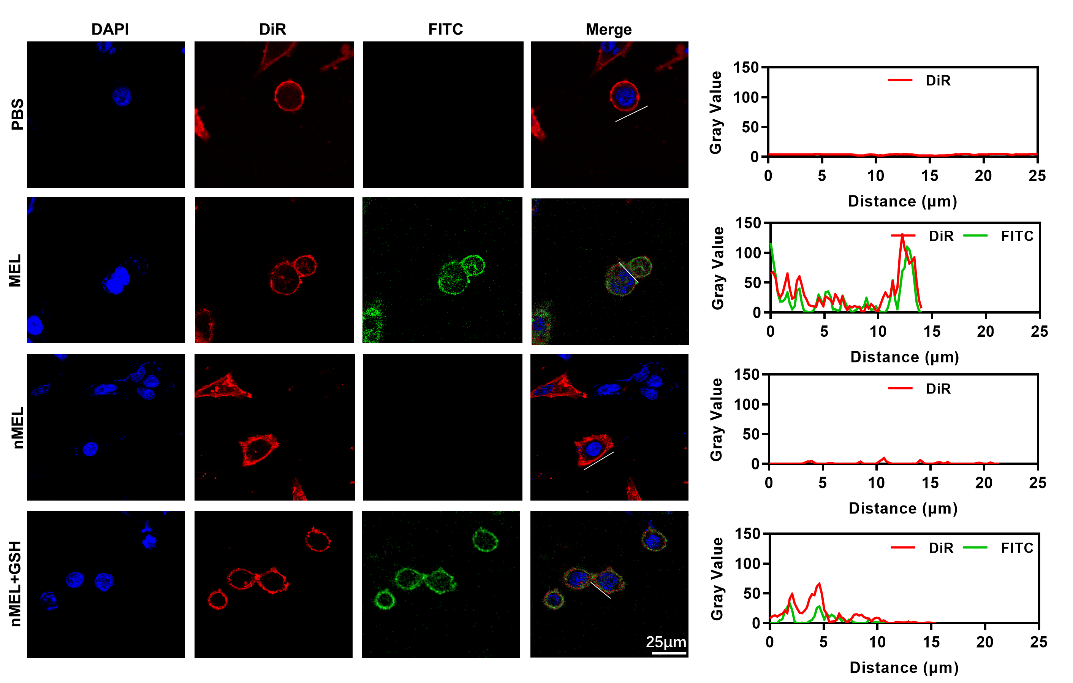


**Figure S1.** Colocalization analysis of membrane-associated double-positive fluorescence signals corresponding to Figure 2c. Representative confocal images of cells incubated with PBS, MEL, nMEL, or nMEL+GSH. Nuclei were stained with DAPI (blue), cell membranes were labeled with DiR (red), and MEL-associated fluorescence was recorded in the FITC channel (green). Merged images are shown to visualize overlap of DiR and FITC signals. For each group, a fluorescence line-scan was performed along the indicated line in the merged image, and the corresponding intensity profiles (gray value vs. distance) for DiR (red) and FITC (green) are plotted to assess spatial colocalization. Scale bar: 25 μm.


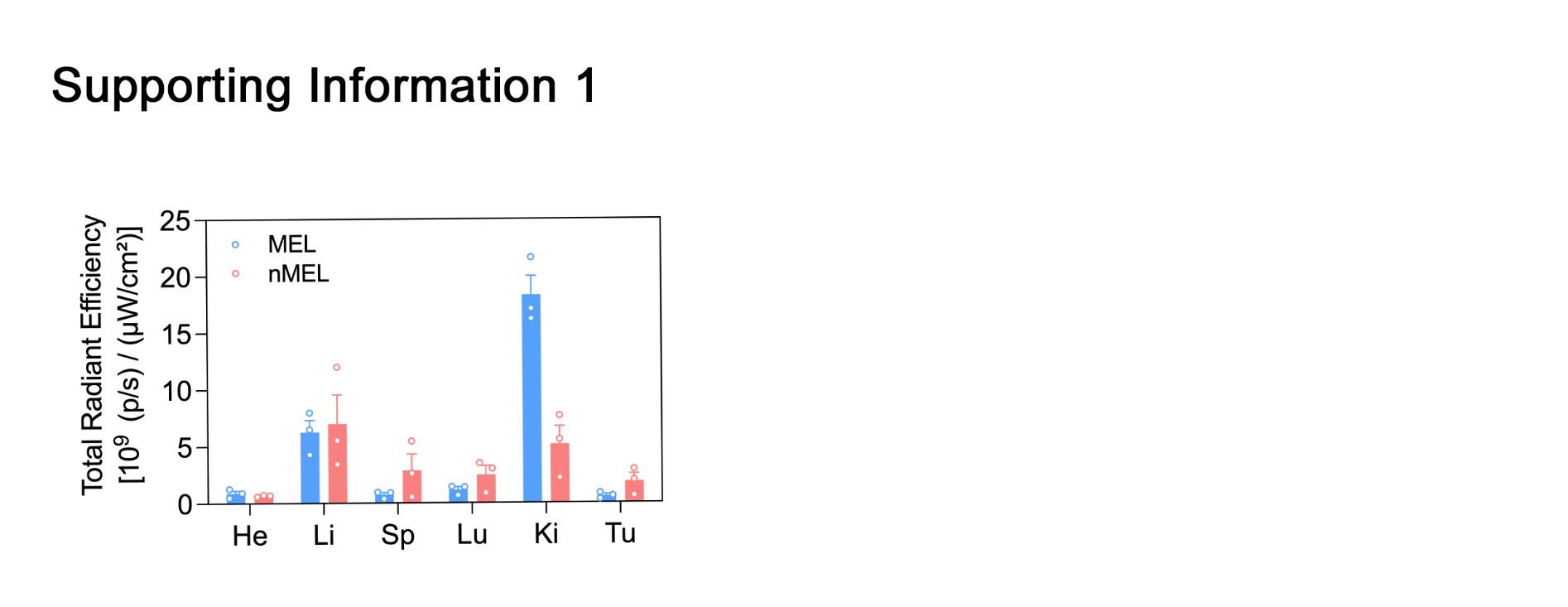


**Figure S2.** Distribution of MEL and nMEL in major tissues quantified by fluorescence intensity. Ex/Em = 680/710 nm.


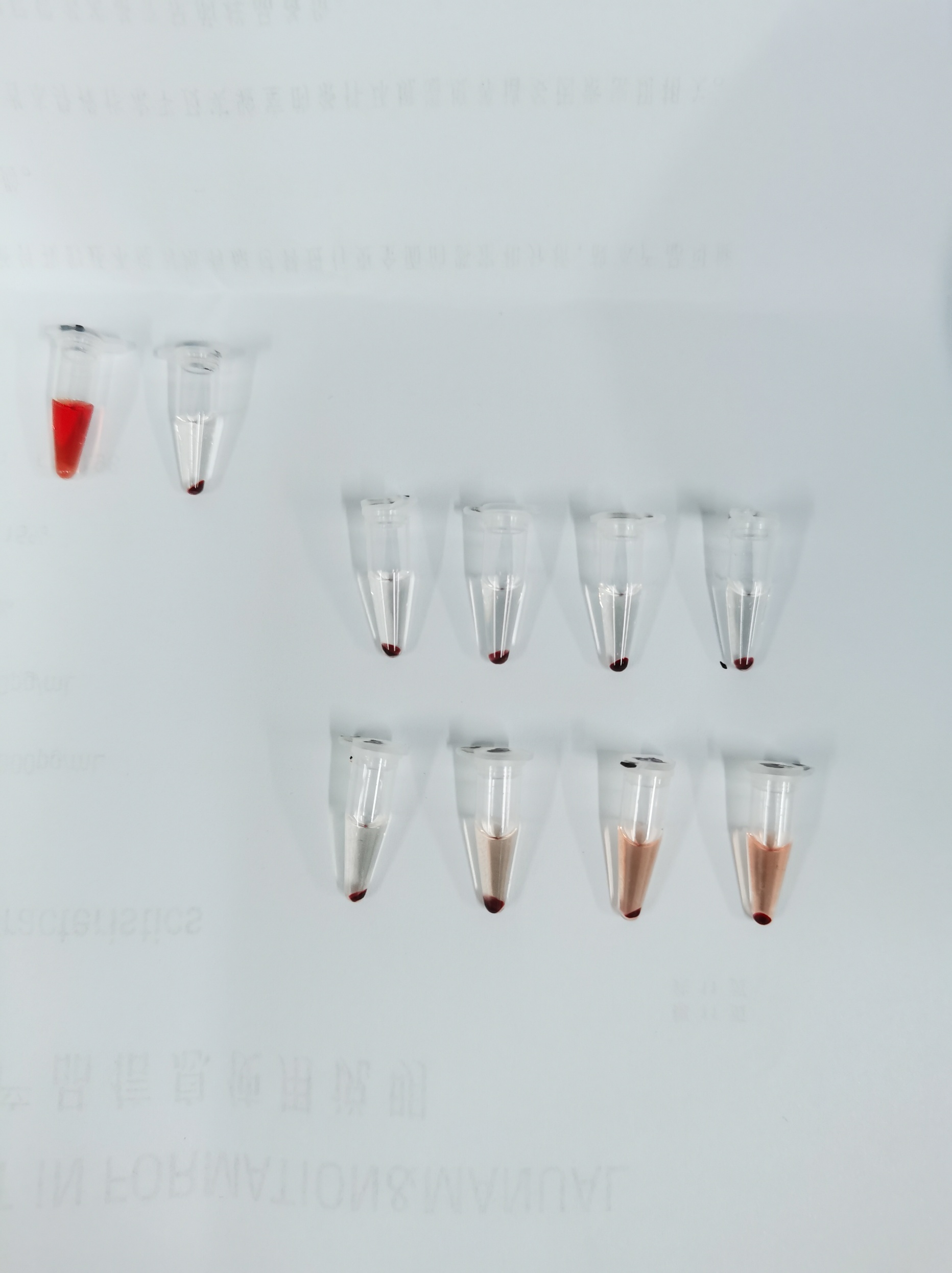


**Figure S3.** Positive (left) and negative(right) controls of Hemolysis assay.


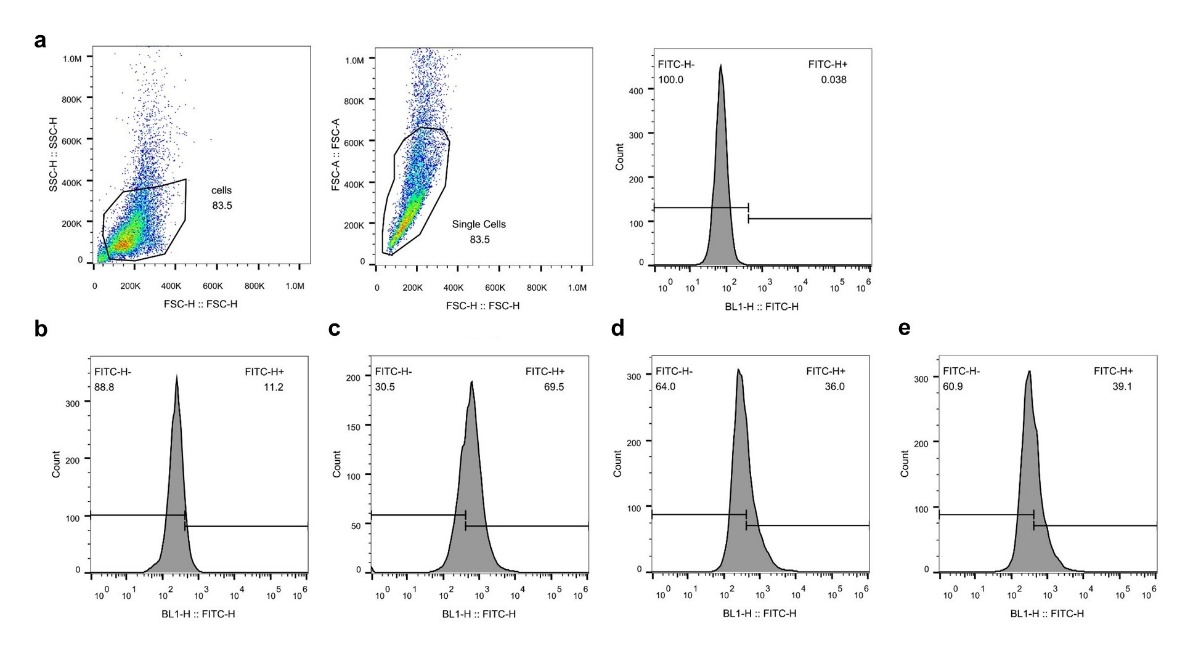


**Figure S4.** Flow-cytometry gating strategy and FITC⁺ thresholding for the cell-surface binding assay (related to Figure 5e–f). (a) Representative gating strategy: events were first gated on FSC-H vs SSC-H to exclude debris/non-cell events (“cells”), followed by FSC-A vs FSC-H to select single cells. FITC fluorescence (BL1-H: FITC-H) was then analyzed on the gated single-cell population, and the FITC⁺ gate was defined using the PBS control and applied identically to all samples. Representative FITC histograms are shown for: (b) nMEL, (c) nMEL-PBA, (d) excess free PBA–blocked (10 mM, 30 min) + nMEL-PBA, and (e) free sialic acid–blocked (10 mM, 30 min) + nMEL-PBA. The percentages of FITC⁻ and FITC⁺ cells are indicated in each panel.


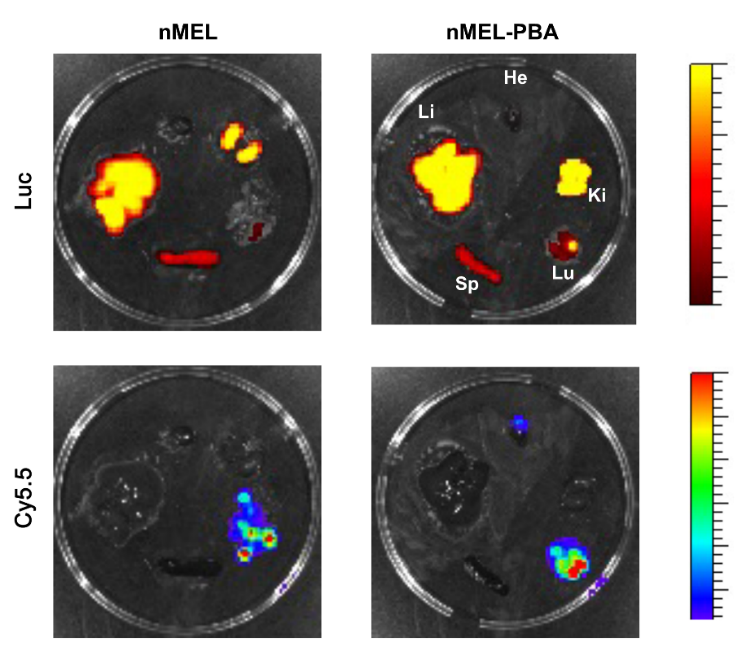


**Figure S5.** Distribution of nMEL and nMEL-PBA in mouse after i.v. injection. A lung metastasis tumor model was established by intravenous injection of 4T1-Luc cells. Tumor signals (Luc), nanocapsule signals (Cy5.5).

**Table S2.** Blood routine examination and Liver and kidney indicators of mice after intravenous injection of MEL and nMEL for 14 days.

| Parameter | Group | Result (mean±SD) | Unit | Reference range |
| --- | --- | --- | --- | --- |
| WBC | PBS | 1.50±0.22 |  |  |
|  | MEL | 2.73±0.84 | 10⁹/L | 0.8–6.8 |
|  | nMEL | 2.10±0.08 |  |  |
| RBC | PBS | 6.79±0.39 |  |  |
|  | MEL | 9.68±0.48 | 10¹²/L | 6.36–9.42 |
|  | nMEL | 8.59±0.55 |  |  |
| HGB | PBS | 95.00±0.00 |  |  |
|  | MEL | 142.67±8.07 | g/L | 110–143 |
|  | nMEL | 119.5±18.88 |  |  |
| MCH | PBS | 14.23±0.74 |  |  |
|  | MEL | 14.62±0.12 | pg | 15.8–19 |
|  | nMEL | 13.23±1.89 |  |  |
| MCHC | PBS | 294.5±13.44 |  |  |
|  | MEL | 302.33±4.89 | g/L | 302–353 |
|  | nMEL | 296.67±31.53 |  |  |
| ALT | PBS | 52.69±14.07 |  |  |
|  | MEL | 118.23±45.93 | U/L | 10.06–96.47 |
|  | nMEL | 59.24±17.32 |  |  |
| ALB | PBS | 29.87±1.09 |  |  |
|  | MEL | 33.22±1.36 | g/L | 21.22–39.15 |
|  | nMEL | 30.28±0.89 |  |  |
| UREA | PBS | 6.00±1.05 |  |  |
|  | MEL | 7.87±0.88 | mmol/L | 3.861–12.41 |
|  | nMEL | 6.74±1.37 |  |  |
| CREA | PBS | 5.74±0.95 |  |  |
|  | MEL | 23.68±3.53 | µmol/L | 10.91–85.09 |
|  | nMEL | 14.52±3.42 |  |  |
| AU | PBS | 32.55±9.98 |  |  |
|  | MEL | 106.65±18.72 | µmol/L | 44.42–224.77 |
|  | nMEL | 86.73±10.45 |  |  |

**Table S3.** Analysis of the survival period of mice with lung metastases.

| Groups | Mean Survival  (Days, mean ± SD) | Median Survival Time (MST, Days) | Percentage increase in life span (ILS, %) |
| --- | --- | --- | --- |
| PBS | 29.00±2.017 | 30 | Control |
| nMEL | 38.83±3.146 | 39 | 30 |
| nMEL-PBA | 43.50±2.729 | 43 | 43.33 |

Data are presented as mean ± SD for Mean Survival (days). Median survival time (MST) was obtained from Kaplan–Meier analysis. Percentage increase in life span (ILS, %) was calculated based on MST: ILS (%)= (MST_treated​_−MST_control​​_) / MST_control​_ ×100

**Reference**

(1) Duffy, C.; Sorolla, A.; Wang, E.; Golden, E.; Woodward, E.; Davern, K.; Ho, D.; Johnstone, E.; Pfleger, K.; Redfern, A.; et al. Honeybee venom and melittin suppress growth factor receptor activation in HER2-enriched and triple-negative breast cancer. *npj Precision Oncology* **2020**, *4* (1), 24.

(2) Lu, L.; Zhang, H.; Zhou, Y.; Lin, J.; Gao, W.; Yang, T.; Jin, J.; Zhang, L.; Nagle, D. G.; Zhang, W.; et al. Polymer chimera of stapled oncolytic peptide coupled with anti-PD-L1 peptide boosts immunotherapy of colorectal cancer. *Theranostics* **2022**, *12* (7), 3456-3473.

(3) Ardeshir, R. A.; Moarefvand, K. Selective apoptosis induction by antimicrobial peptide TP4 in MCF-7 breast cancer cells. *Scientific Reports* **2025**, *15* (1), 15061.

(4) Ramos-Martín, F.; D’Amelio, N. Molecular Basis of the Anticancer and Antibacterial Properties of CecropinXJ Peptide: An In Silico Study. *International Journal of Molecular Sciences*, **2021**, *22* (2), 691.

(5) Kang, Z.; Wang, C.; Zhang, Z.; Liu, Q.; Zheng, Y.; Zhao, Y.; Pan, Z.; Li, Q.; Shi, L.; Liu, Y. Spatial Distribution Control of Antimicrobial Peptides through a Novel Polymeric Carrier for Safe and Efficient Cancer Treatment. *Advanced Materials* **2022**, *34* (23), 2201945.

(6) Chen, T.; Wang, Y.; Yang, Y.; Yu, K.; Cao, X.; Su, F.; Xu, H.; Peng, Y.; Hu, Y.; Qian, F.; et al. Gramicidin inhibits human gastric cancer cell proliferation, cell cycle and induced apoptosis. *Biological Research* **2019**, *52* (1), 57.

(7) Hashem, S.; Bhat, A. A.; Nisar, S.; Uddin, S.; Merhi, M.; Mateo, J. M.; Prabhu, K. S.; Soubra, L.; Dos Santos Silva, C. A.; Benko-Iseppon, A. M.; et al. Engineered Anti-Microbial Peptides Inhibit Cell Viability, Promote Apoptosis, and Induce Cell Cycle Arrest in SW620 Human Colon Adenocarcinoma Cells. *Current Protein & Peptide Science* **2025**, *26* (7), 570-584.

(8) Benfield, A. H.; Vernen, F.; Young, R. S. E.; Nadal-Bufí, F.; Lamb, H.; Hammerlindl, H.; Craik, D. J.; Schaider, H.; Lawrence, N.; Blanksby, S. J.; et al. Cyclic tachyplesin I kills proliferative, non-proliferative and drug-resistant melanoma cells without inducing resistance. *Pharmacological Research* **2024**, *207*, 107298.
